# Downbeat nystagmus: a clinical and pathophysiological review

**DOI:** 10.3389/fneur.2024.1394859

**Published:** 2024-05-24

**Authors:** Vincenzo Marcelli, Beatrice Giannoni, Giampiero Volpe, Mario Faralli, Anna Rita Fetoni, Vito E. Pettorossi

**Affiliations:** ^1^Audiology and Vestibology Unit, Department of ENT, Ospedale del Mare, ASL Napoli 1 Centro, Napoli, Italy; ^2^Department of Neuroscience, Reproductive Science and Dentistry, Section of Audiology, University of Naples ‘’Federico II’’, Napoli, Italy; ^3^Department of Neuroscience, Psychology, Drug’s Area and Child’s Health, University of Florence, Florence, Italy; ^4^Department of Neurology, Ospedale San Luca di Vallo della Lucania, ASL Salerno, Salerno, Italy; ^5^Department of ENT, University of Perugia, Perugia, Italy; ^6^Department of Medicine and Surgery, University of Perugia, Perugia, Italy

**Keywords:** downbeat nystagmus, downbeat nystagmus syndrome, vertical reflex asymmetry, neural integrator, central neurological disorders

## Abstract

Downbeat nystagmus (DBN) is a neuro-otological finding frequently encountered by clinicians dealing with patients with vertigo. Since DBN is a finding that should be understood because of central vestibular dysfunction, it is necessary to know how to frame it promptly to suggest the correct diagnostic-therapeutic pathway to the patient. As knowledge of its pathophysiology has progressed, the importance of this clinical sign has been increasingly understood. At the same time, clinical diagnostic knowledge has increased, and it has been recognized that this sign may occur sporadically or in association with others within defined clinical syndromes. Thus, in many cases, different therapeutic solutions have become possible. In our work, we have attempted to systematize current knowledge about the origin of this finding, the clinical presentation and current treatment options, to provide an overview that can be used at different levels, from the general practitioner to the specialist neurologist or neurotologist.

## Introduction

Downbeat nystagmus (DBN) is the most common form of acquired central nystagmus (1) and is therefore a very frequent finding in daily clinical practice in both the primary care and outpatient settings, from the first to third level of complexity.

Since the initial descriptions, there have been many acquisitions that have led to an ever-greater understanding of the pathophysiological mechanisms underlying DBN, and there have been many implications that these findings have had in both the clinical and therapeutic fields. Despite the large number of publications and some recent reviews, a comprehensive view of the clinical and experimental evidence together with a perspective of pathophysiological interpretation for the different aspects of DBN seems necessary and useful also for clinical purposes. In this review, we trace the steps from the first descriptions of vertical nystagmus to the current “downbeat nystagmus syndrome” (DBNS). Thereafter, we will describe what is known about the clinical characteristics of DBN, we will make an overall overview of the various pathophysiological mechanisms that underlie it, and we will describe the central nervous structures of the central nervous structures whose alterations underlie the signs and symptoms.

Ultimately, our intention was to provide a unified view of this syndrome in terms of etiology, of clinical and nystagmus features, associated oto-neurological signs, and genetic aspects, useful for the identification of the disorder and, sometimes, for the rapid establishment of the most appropriate diagnostic and therapeutic procedure.

### First descriptions of vertical nystagmus

Vertical nystagmus received little attention in the early medical literature. For instance, Boehm in 1857 (2) and Fuchs in 1908 (3) did not even discuss vertical nystagmus in their papers. In 1921, Wilbrand and Saenger (4) started to note that vertical nystagmus could be produced in experimental animals by cutting the anterior semicircular canal (SCC) in the labyrinth and found it in some patients with severe bilateral visual loss, drug toxicity (barbiturates and quinine), multiple sclerosis, spasmus nutans, lesions of the pons and medulla (tuberculoma and meningioma), and encephalitis lethargica. In 1950, Berens and McAlpine (5) stated: “Bilateral vertical or mixed movements of nystagmus are rare…. Unfortunately, the direction of the nystagmus has no diagnostic value, and the main clinical problem is the differential diagnosis of whether it is peripheral or central.” On the contrary, Walsh (6) reported in his 1947 review that purely vertical nystagmus was “central or cerebellar,” describing it in two patients with Arnold-Chiari malformation and in one subject affected by cerebellar ataxia, bilateral pyramidal tract involvement and hydrocephalus. However, Walsh does not describe the characteristics of the nystagmus. Although all these authors recognized vertical nystagmus as a sign of central nervous system lesion, they specifically never described its direction.

### First descriptions of downbeat nystagmus, its causes and the effects of eccentric gaze and head-body position

Cogan and Barrows (7) described a vertical nystagmus in nine patients with platybasia or Arnold-Chiari malformation; such a sign was present in the primary gaze position in eight subjects of his series. For the first time, the authors described the direction of the nystagmus; two patients had DBN. In one patient, DBN was present in central gaze and decreased during reading (convergence). In the other, DBN was present only in lateral gaze. All but one of the nine patients had symptoms and signs due to compression of the brainstem and/or cerebellum. Cogan (8) published the first study to systematically describe the characteristics, causes and localization of DBN. In particular, the effects of eccentric gaze and head or body position were evaluated. The most common symptoms associated with DBN were found to be oscillopsia and gait difficulty due to ataxia or corticospinal tract impairment. The radiological and surgical findings led Cogan to suggest that lesions causing DBN may be localized in the cerebellum or in the lower brainstem. After this historical paper numerous authors (9–14) confirmed that cerebellar lesions such as Arnold-Chiari malformations, hereditary and sporadic cerebellar degenerations, and other acquired cerebellar degenerations (alcoholic, paraneoplastic and anoxic) can cause DBN. It is important to note that almost all researchers have also already described some peculiarities of DBN, such as the increase in the velocity of its slow component in lateral gaze and in certain positions of the head.

Experimental studies in animals have also confirmed that some lesions usually confined to the caudal cerebellum can produce DBN (15–20).

### Evidence of downbeat nystagmus syndrome with nystagmus, perceptual and balance alteration

The association of DBN with oscillopsia, impaired environmental motion perception, mislocation of objects in the direction of the slow phase of nystagmus and an imbalance that causes the body to oscillate predominantly in the antero-posterior direction, constitute a true syndrome called “Downbeat Nystagmus Syndrome” (DBNS) characterized by a tonic vestibular imbalance of neural circuits in the sagittal plane due to damage to central neural pathways.

## Characteristics of downbeat nystagmus (DBN)

DBN is usually clearly visible in the primary gaze position, but it can be present, alternatively, or simultaneously with this state, even in the eccentric position of gaze. The most important feature of DBN is its strong gravitational dependence. Convergence and lateral gaze can increase its slow phase velocity (SPV) or even cause nystagmus. As DBN most often follows Alexander’s law (21–23) downward gaze makes the nystagmus more apparent. DBN is not inhibited by visual fixation (24–26).

Other conditions such as hyperventilation, head shaking, and visual fixation can affect the quality of DBN and sometimes change its direction. Very rarely, DBN occurs in combination with internuclear ophthalmoplegia. In this case it is disconjugate, with a vertical component in one eye and a torsional component in the other (27).

The slow phase of nystagmus is usually linear but may show either a negative or positive exponential trend in the presence of brainstem neural integrator dysfunction.

Because DBN is caused by an imbalance of the reflex in the vertical plane, the eye movement has a torsional component in lateral gaze (Table 1).

**Table 1:** Cerebellar and extracerebellar sites of lesion and the mechanisms underlying them.

Cerebellar flocculus/paraflocculus
Cerebellar vermis
Nodulus/Uvula
Dorsal larger cell Y-group (Yd) of the Y-group nucleus
Otolith-ocular circuit
Vertical neural integrator
Floor of the fourth ventricle
Pons
Medulla oblongata

As mentioned above, the DBN can be observed in primary (1) or eccentric eye position (2).

*1) Nystagmus that is present in primary gaze position*.

Regarding the nystagmus present in the primary gaze position, we can recognize it in two different components:

a) A gravity-independent (GI) direction-fixed, chin-directed DBN;
b) a gravity-dependent (GD) direction-changing vertical nystagmus.

The effect that gravity has on vertical drift and VOR through stimulation of otolith receptors is well documented (28). In fact, even normal subjects show vertical drift/nystagmus in the dark which is systematically modulated by both the position of the eyes in the orbit and by the orientation of the head in space (29, 30) In the presence of a cerebellar lesion, this directional influence of gravity is greatly increased (31–33) and electrophysiological studies demonstrated that both the flocculus (34, 35) and the nodulus (36) modulate otolith-ocular reflexes. The role of the cerebellum in controlling the modulation exerted by gravity on the DBN has been clarified by the administration of 3,4-diaminopyridine (3,4-DAP), a drug which acts on the Purkinje cells. Indeed, it has been demonstrated that the drug affects the cerebellar gravity-dependence of nystagmus in different sagittal head positions but does not alter nystagmus in the standing position (37). The modulation of the GD component in the sagittal plane could be due to disinhibition of the canalar reflex in the same plane, modulated by the macular input. As a result of this modulation, in the prone position, as in head flexion, the GD component is directed upwards, whereas in the supine position, as in head extension, the GD component is directed downwards. Due to the presence of these two components, the direction and SPV of the nystagmus are determined by the combination of a fixed GI component (always directed upwards) and a variable vertically directed GD component. In the *prone position*, the SPV of the nystagmus has the highest values: the GI component directed upwards is indeed added to the GD component directed in the same direction, which has the maximum intensity in this position. On the other hand, the SPV is lowest in the *supine position*: the GD component shows its maximum downward drift, counteracting the upward drift of the GI component. The opposite direction of the vertical drift caused by the two components is therefore responsible for a very low SPV in the supine position. In the *upright position*, the GD component is close to zero, so the vertical drift of the eyes in this position is mainly due to the GI component. In the *head hanging position*, the SPV increased again compared to the supine position because the downward GD component decreased, probably due to the different position of the head and/or the compression force on neural structures, as in Arnold-Chiari disease.

*2) DBN that is present only in eccentric gaze on vertical plane*.

In such a condition, an orbital-position-dependent effect is present that is likely due to a damage of the vertical neural integrator (VNI) is present. Then, when looking downwards, the result will be a slow centripetal drift of the eyes, which in turn will produce a retinal slip which will generate a rapid eye movement to recover gaze eccentricity, ultimately producing or enhancing the DBN. In this case, the slow phase of the DBN is not linear but either negative or positive exponential.

Although DBN in primary gaze is a sign of CNS dysfunction, it is not rarely possible to observe a positional down beat nystagmus (pDBN), in the presence of which a differential diagnosis with anterior canal BPPV or apogeotropic variant of PSC BPPV is necessary (38–42).

## Pathophysiology for DBN

To understand how DBN can develop, it is necessary to consider the existence of an innate asymmetry for the vestibulo-oculomotor vertical reflexes in favor of upward eye movements both in the peripheral apparatus and in the central circuits. Such an imbalance is probably designed to counteract the gravitational forces that naturally oppose upward eye, head, and body movement.

### Evidence for vertical reflex asymmetry

The first evidence for an upward reflex preponderance comes from the observation that in cats, dogs, monkeys and humans, in which DB has been shown to be more difficult to suppress than up beat nystagmus (43–46), there is a clear tendency for upward ocular drift in darkness, which increases when the head is tilted away from the upright position due to an otolith-driven central mechanism (47). On the other hand, it has been shown (48, 49) that optokinetic nystagmus and optokinetic afternystagmus as well as nystagmus induced by vertical rotation predominate in the downward direction. In fact, the time constant for DBN is about 15 s, which is like that of horizontal nystagmus, whereas for upbeat nystagmus it is about 8 s. This intrinsic time constant asymmetry is also modulated by the otolith end organs, which suppress the velocity storage mechanism for downward directed nystagmus in the upright position and enhance it during downward low frequencies head movements (50). Further confirming the asymmetry in the sagittal plane and the role of the macula, in the upright subject, is that the forward pitch sensation during the surround movement that produced the downward OKN is stronger than the backward pitch sensation associated with upward OKN, and this effect is significantly enhanced in the side lateral position (51).

### Peripheral asymmetry of the vertical vestibulo-ocular reflex

**Figure 1:**
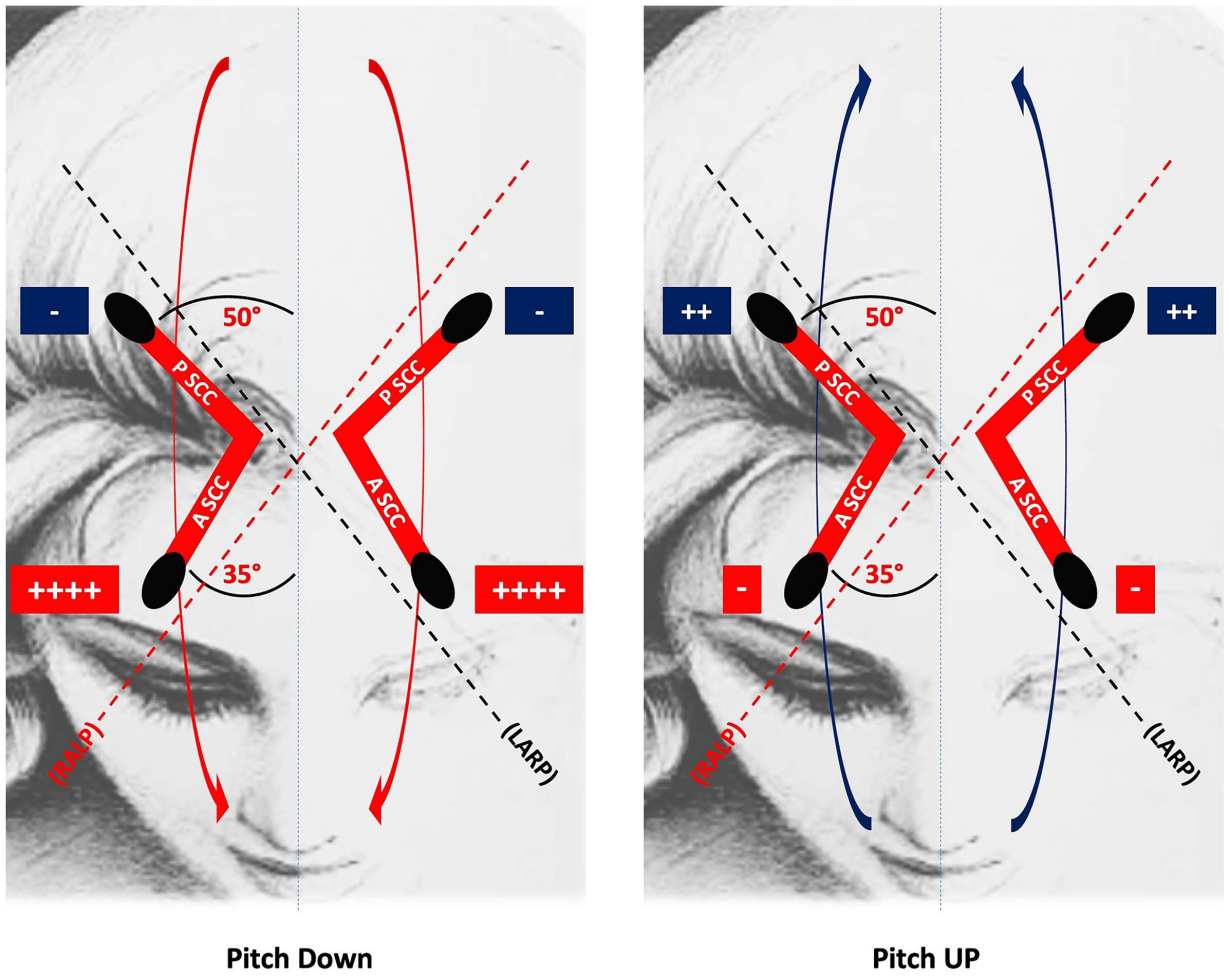
The upward eye response to downward head rotation is greater than the downward eye response to upward head rotation because the anterior SCC is more aligned with the sagittal plane than the posterior SCC: thus, the ampullofugal, excitatory response of the anterior SCC cupula to downward head rotation is greater than the homologous response of the posterior SCC cupula to upward head rotation. RALP, right anterior, left posterior; LARP, left anterior, right posterior; PSCC, posterior semicircular canal; ASCC, anterior semicircular canal.

**Figure 2:**
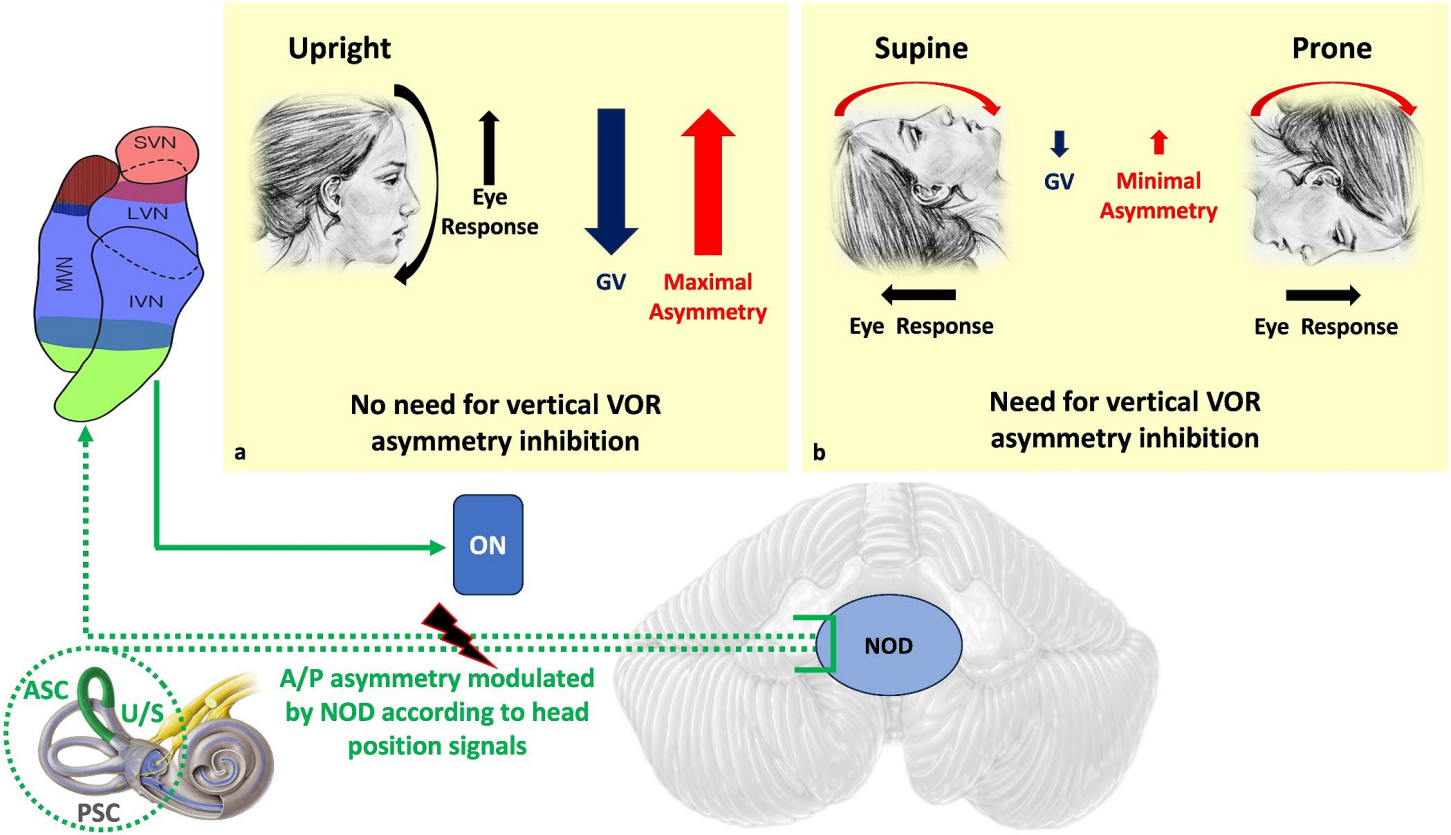
Dashed green is the NOD circuit that modulates the A/P asymmetry of the vertical VOR and upward slow phase due to different gravitational vector effects on the macula depending on head position. In the upright position (a), the preponderance of upward eye responses is needed to counteract the effect of gravity, so peripheral asymmetry should not be inhibited. In the supine and prone positions (b), where gravity no longer opposes movement, the natural asymmetry of the VOR is no longer necessary. Therefore, the asymmetry of the vertical VOR should be inhibited. Direct NOD lesion in humans produces positional DBN. ASC, Anterior semicircular canal; PSC, Posterior semicircular canal; U/S, Utricle/Sacculus; ON, Oculomotor nuclei; A/P, Anterior/Posterior; NOD, Nodulus/Uvula; GV, gravitational vector.

At the peripheral level, the preponderance of upward eye movements is supported by the anatomo-physiological properties of the vertical SCCs (34). Indeed, the anterior SCCs are more aligned with the sagittal plane than the posterior canals. Therefore, the ampullofugal stimulus, which is excitatory for the vertical SCCs, excites the cupula of the anterior SCCs better during downward head rotation than the cupula of the posterior SCCs during upward head rotation. Thus, the vertical head rotation signal is naturally asymmetric, manifesting a preponderance during downward rotations (Figure 1). This state of asymmetry must be subject to central inhibitory control. Moreover, the antigravitational effects must be modulated according to the action of gravity and therefore according to the different positions of the head in space (Figure 2). Consequently, if these central inhibitory control areas are damaged, a nystagmus with a slow upward phase and a fast downward phase (DBN) can be induced.

### Asymmetry of the centers controlling the vertical reflex

Regarding the circuits that control vertical asymmetry, it has been shown that some cerebellar and extra-cerebellar structures are dedicated to controlling the activity of VOR responses in the vertical plane. These areas have been identified in the flocculus/paraflocculus, nodulus, vermis and some brainstem areas.

*1. Role of the Flocculus/Paraflocculus*.

a. *Cerebellar Purkinje cells of the rostral and caudal zones of the flocculus (F/PF)*. These cells are involved in the inhibitory control of upward vertical rotational VOR, projecting to superior vestibular nucleus (52–55). In fact, their lesion results in relative hyperactivity of the anterior SCCs circuit (14, 34, 55–57), which, in turn, generates a DBN. Such a hyperactivity is confirmed by the finding of an increased upward VOR gain when performing a downward head impulse test (14, 58–60). Since the F/PF is involved in both the generation and in control of smooth pursuit and the control of ocular eccentricity, a lesion involving these structures will also result in impaired vertical smooth pursuit and gaze-evoked nystagmus (GEN). Downbeat nystagmus seen with flocculus/paraflocculus lesions cannot be suppressed with visual fixation.
b. *Cerebellar flocculus/paraflocculus (F/PF) and the dorsal larger cell Y-group (Yd) of the Y-group nucleus*. The Y-group nucleus of the vestibular nuclear complex is a well-defined nucleus in the brainstem. A subpopulation of the Y-group nucleus, the dorsal larger Y group (Yd), contains a large population of excitatory neurons that target up gaze motoneurons in the contralateral oculomotor nucleus and GABAergic inhibitory neurons that target motoneurons in the ipsilateral oculomotor and trochlear nuclei (58–61). The Y-group nucleus receives a disynaptic excitatory input from the ipsilateral anterior and posterior SCCs via neurons in the superior vestibular nucleus and caudal medial vestibular nucleus (MVN), and a strong inhibitory input from Purkinje cells of the F/PF (62–65). A lesion of the Purkinje cells results in disinhibition of the Y-group cells, which leads to upward sliding of the eyes, and induces DBN.

2. *Role of the Nodulus/Uvula and otolith*.

It has been shown that almost half of the Purkinje cells sensitive to vertical SCCs activation in the nodulus/uvula (NOD) are modulated by otolith signals (28, 36, 56, 58, 66, 67), that signals from SCCs and otolith receptors reach specific areas of the NU where they are integrated (68), and that the same signals often converge on a single Purkinje cell. It is also interesting to note that the afferent signals from anterior and posterior SCCs project in different areas. In this way, the two circuits have the possibility of separate modulation for movements in both the frontal and sagittal plane, to influence differently the preponderance of the peripheral vestibular signals. Such convergence of inputs therefore allows the control of the inhibitory SCCs to be modulated by head position signals.

In particular, the natural preponderance of responses inherent to the circuitry of the anterior SCCs can be modulated during downward head movement depending on how the force of gravity opposes the eye and head responses (Figure 2).

The role of the nodule/uvula is further supported by the observation of increased intensity of the DBN following neurosurgical aspiration of these structures in monkeys (69) and the loss of orientation of the horizontal afternystagmus, which also indicates the orientational role of the nodule in ocular responses (see “Associated oto-neurological signs” section).

In addition, it has been shown that the velocity of the slow phase of head-down nystagmus is no longer inhibited after nodule removal (66, 70, 71) and that direct NOD lesion in humans produces positional DBN (72).

In contrast to the downbeat nystagmus seen in flocculus/paraflocculus lesions, the nystagmus due to nodulus/uvula lesions can be suppressed by visual fixation, is only seen in the dark, and the slow phase velocity is not increased in lateral gaze or decreased in up gaze. These findings suggest that downbeat nystagmus in nodulus/uvula lesions is unlikely to be related to an abnormal vertical ocular motor integrator, but rather to a central vestibular imbalance, possibly in the otolith-ocular pathways that mediate the vertical translational VOR (69).

In this way, direct damage to the otolith-ocular circuit can also cause DBN (58). In humans, DBN has been shown to be induced by static head tilt and linear acceleration independent of the canal planes and corresponding to the static and dynamic components of otolith function (73, 74). Changes in static head orientation in the sagittal plane are known to influence DBN through otolith modulation (30, 75). The role of otolith inputs is also demonstrated during oscillation about the upright position: the gain of the upward VOR increases and the gain of the downward VOR decreases, with a degree of VOR asymmetry greater than would be expected from a simple summation of spontaneous nystagmus with normal SCCs reflexes (58).

In addition, mutant mice with calcium P/Q channelopathy, which lose many of the critical electrical properties of the cerebellar Purkinje cell dendrite, show hyperactivity of the otolith-ocular reflexes (76) and static ocular hyperdeviation, which is the murine equivalent of the upward velocity bias in DBN (77). As mentioned above, the imbalance of the macular-canal pathways would potentially explain the modulation of nystagmus by the gravitational vectors in most, if not all, patients, and damage to these structures would therefore produce a disinhibition of the macular-canal interaction (21, 37, 58–60, 72, 78–83). The role of macular input is further demonstrated by the change in DBN SPV induced by convergence, a reflex that is modulated by the macula.

3. *Role of cerebellar areas implied in vertical smooth pursuit: flocculus/paraflocculus, vermis Nodulus/Uvula*

a. *Flocculus/paraflocculus*. A central imbalance in vertical smooth pursuit (SP) tone has been considered as a possible origin of DBN due to impairment of Purkinje cells of the F/PF and its complex pathway (medial and superior VN, dorsolateral pontine nuclei, ocular motor dorsal vermis, fastigial nucleus, nucleus reticularis tegmenti pontis, prepositus nuclei). In particular, the just reported bias of Purkinje cells to discharge with downward movements could explain the asymmetric vertical pursuit in patients with downbeat nystagmus. A lesion involving such cells would result in upward eye gliding and consequent DBN.
b. *Vermis*. In addition to the Purkinje cells of the flocculus, there is another Purkinje population in the dorsal vermis (lobules VI, VII) that plays a critical role in vertical SP eye movements, firing more effectively during downward than upward smooth pursuit, hence the term “downward directed floccular cells” (60, 78, 84, 85). Therefore, also a lesion involving such cells would result in upward eye gliding and consequent DBN.
c. *Nodulus/uvula*. A critical role for the NOD in vertical pursuit, particularly in sustained downward pursuit, has been demonstrated, and in monkeys lesion of the NOD increased spontaneous upward eye drift in the dark. This finding confirms a role for the NOD in eye fixation and in the generation of downbeat nystagmus (83).

4. *Role of extra-cerebellar areas: vertical neural integrator, floor of the fourth ventricle, medulla oblongata, pons, lower motor neurons*

a) The *neural integrator for vertical eye movements* is a complex neural network whose structures are largely located in the midbrain and project indirectly, from the interstitial nucleus of Cajal [via the paramedian tract nucleus (PMT)], or directly, via the PMT, to the FL/PFL. The VNI receives velocity commands from all the conjugate eye movements systems and converts them into a position command suitable for maintaining vertical gaze eccentricity once it has been reached if this is required. Damage to these structures can lead to DBN, as has been shown in non-human primates with lesions of the PMT (86), which determined a reduced FL/PFL input, a reduced PC output and, finally, a hypofunction of the FL/PFL. A lesion of this neural network can also alter the integrator time constant and the intrinsic orientation coordinate system, and thus still result in DBN as the eyes are unable to maintain eye position correctly and rapid reset phases are triggered to regain downward position (21, 55, 81, 86). In addition, the VNI may become “leaky” or “unstable,” characteristically modifying the DBN intensity:

a. a “leaky” NI determines a velocity decreasing slow-phase waveforms and nystagmus intensity which increases looking in the direction of the fast phase.
b. an “unstable” NI determines a velocity increasing slow-phase waveforms and nystagmus intensity which increases looking in the direction of the slow phase.

b) The *floor of the fourth ventricle* contains the vertical VOR pathways starting from the posterior SCCs, whose input reaches the medial and lateral vestibular nuclei. Injury of this pathway will result in the loss of the tonic activity of the posterior SCCs, resulting in upward ocular drift and DBN (87). The possible involvement of the medial longitudinal fasciculus, traveling in a craniocaudal direction near the midline within the tegmentum of the midbrain and dorsal pons immediately ventral to the cerebral aqueduct and fourth ventricle, justifies the possible associated internuclear ophthalmoplegia.
c) The *medulla oblongata* contains the paramedian tract neuron (PMT) cells, which exert a physiological tonic excitation on floccular Purkinje cells. Injury of these cells reduces the activity of floccular Purkinje cells, which in turn reduces the inhibition of secondary vestibular nucleus neurons that receive input from the anterior SCCs. This disinhibition induces upward ocular drift and thus the production of a DBN (88, 89).
d) The *Pons* contains the nucleus hypoglossi prepositus (NPH), that is the basic structure of horizontal neural integrator (HNI). An injury to the HNI may also result in a DBN. Experimental animal study and case reports have shown that NPH dysfunction can also results in vertical ocular deviations, suggesting some overlap between horizontal and vertical gaze control (90). Of course, the proximity between the NPH and MVN, approximately 0.9–1.1 mm apart in felines (91, 92), does not exclude that the lesion may also affect the VMN, in addition to that of the neural integrator, which would be the true cause of DBN.
e) *Lower motor neurons*. Finally, it must also be remembered the existence of an association between DBN and the lower motor neuron disease, whose etiology remains unknown (93, 94).

### Effects of ocular convergence and ocular deviation on DBN

Ocular convergence and ocular lateral deviation have been reported to induce or facilitate DBN (1, 57, 95). A possible explanation for these effects is the presence of a cross-coupling mechanism between the circuits of convergence and lateral gaze deviation and vertical eye movements. It is likely that lesions producing DBN can also destroy these separate circuits. Regarding convergence, it is well known that the vertical otolith-ocular reflex should depend on the distance between the target and the observer, with an increase in the reflex gain in the presence of a near object to be observed (96). Thus, the reflex is facilitated along with the activation of vergence. In DBN patients, the two interrelated mechanisms of vergence and gain enhancement are cross-coupled. Therefore, vergence could enhance any vestibular reflex, but if there is an imbalance in the vertical circuits, vergence could reveal or induce DBN. A similar cross-coupling may be responsible for the occurrence of DBN with lateral eye deviation. The lateral deviation of the eye could be interpreted by the central nervous system because of a lateral displacement of the head, activating the translational macular reflex in the vertical plane, making the eyes sliding upwards (68, 73, 97, 98). Because of the damage of the center controlling the otolithic networks, cross coupling between horizontal and vertical otolithic reflexes may occur, inducing increased excitability in the vertical reflex. Therefore, in presence of an imbalance in the vertical eye movement control, this control excitability would facilitate the DBN (57).

**Figure 3:**
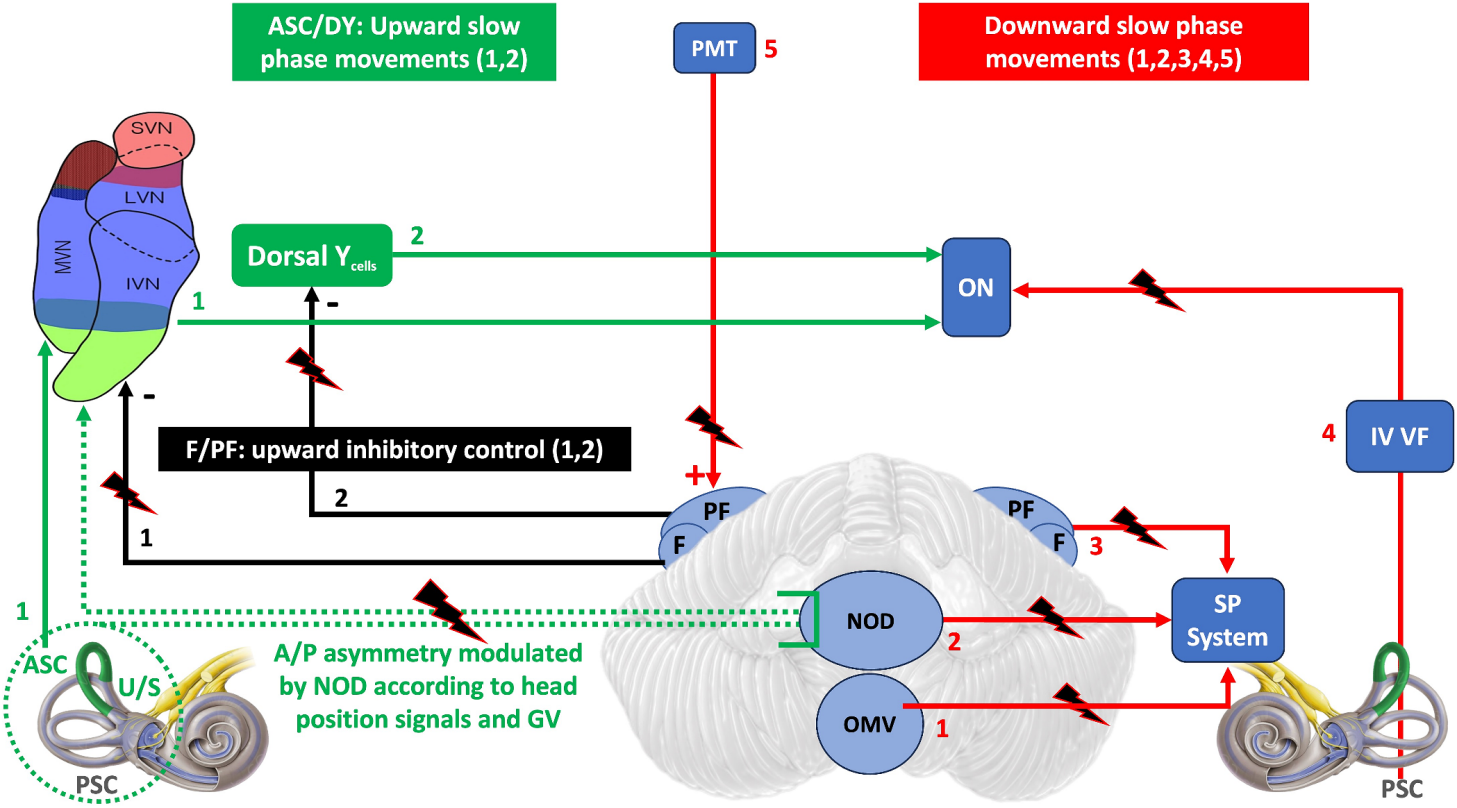
The diagram is a schematic representation of the neural areas involved in the control of vertical eye movements and the possible locations of lesions that may result in DBN. In green are the areas directly responsible for generating an upward slow phase: ASC and dorsal Y. In black are the FL/PF inhibitory efferents to the vestibular nuclei that modulate the upward slow phase. In red are the areas destined to generate a downward slow phase: 1,2,3,4 directly. 5 (PMT) indirectly, because by exciting the F/PF, it favors the inhibition of the upward slow phase and thus the generation of a downward slow phase. Dashed green as in Figure 2. ASC, Anterior semicircular canal; PSC, Posterior semicircular canal; U/S, Utricle/Sacculus; A/P, Anterior/Posterior; GV, Gravitational vector; F/FP, Flocculus/Paraflocculus; DY, Dorsal Y cells; PMT, paramedian tract nucleus; ON, Oculomotor nuclei; OMV, Oculomotor vermis; NOD, Nodulus/Uvula; SP, Smooth Pursuit; IV VF, Floor of VI ventricle.

Figure 3 summarizes the possible cerebellar and extracerebellar sites of lesion and the mechanisms underlying them.

### Circadian modulation of DBN

The DBN decreases spontaneously during the day when the subject remains in an upright position and in darkness. Indeed, the slow phase velocity (SPV) decreases by about 50% during the day and remains constant in the afternoon (99). On the other hand, the SPV measured in the upright position is lower after resting in the upright position than after resting in the supine or prone position. This effect does not depend on fixation, eye position or body position. Several hypotheses have been proposed to account for the possible role of otolith signals in stabilizing vertical circuits of the VOR during prolonged upright rest (100, 101). Other influences can be considered, such as the role of extraocular proprioception (102), efference copy (103, 104), sleep inertia, which is a transient state of reduced arousal that occurs immediately after awakening from sleep (105–107) and has a negative influence on Purkinje cell function. Finally, the effect of resting for 2 h in darkness was also considered, which significantly lowered the post-rest DBN, in contrast to the effect of resting in light (108).

## Symptoms of downbeat nystagmus syndrome

The symptomatology of Downbeat Nystagmus Syndrome (DBNS) is essentially characterized by oscillopsia and motion illusion, diplopia, internuclear ophthalmoplegia (INO) and/or skew deviation, sagittal imbalance, and increased risk of falls.

Oscillopsia (109) is the most disabling symptom. It is caused by the shifting of images on the retina because of the involuntary slow phase of nystagmus. In the absence of an efferent copy, which is generated only in the presence of a voluntary movement, the pathological displacement of the eyes creates an illusion of movement of the visual scene. The illusion is less intense compared to the magnitude of the slow-phase eye movements, presumably due to a reduction in the sensitivity of visual motion perception (110), which is useful to mitigate the annoyance of illusory oscillations. Indeed, the estimated ratio between the illusory motion and the slow-phase angular velocity (SPAV) is 0.371. However, this perceptual reduction also reduces the visual feedback necessary for motion control, thus altering responses to movement and altering foot trajectory and positioning during walking, ultimately resulting in additional imbalance (111, 112).

On the other hand, the same motion illusion due to oscillopsia generates a compensatory vestibulo-spinal reflex (VSR) that contributes to the imbalance. In fact, under physiological conditions, flexion of the head and/or trunk activates the vertical VOR, responsible for an upward slow phase, and the vestibulo-collic and vestibulo-spinal reflexes, responsible for an increase in posterior spinal muscle tone necessary to balance the flexion.

Under pathological conditions, the tonic asymmetry generated not by the subject’s movement but by the central vestibular pathways causes an upward eye movement and generates an antero-pulsion misperception characterized by an illusory head and/or body flexion. The resulting postural compensation leads to an increase in posterior spinal muscle tone to compensate for the illusory flexion, which is characterized by retropulsion and postural oscillations in the antero-posterior direction. These effects are not dependent on vision, as they have been demonstrated even with the eyes closed (113). In conclusion, the postural oscillations present with eyes open are a direct result of several mechanisms induced by the lesion and/or an indirect consequence of the illusory perception of body displacement.

In addition, is typical of the DBS a peculiar gait disturbance characterized by a reduced step speed, a shorter swing phase, and a longer double-support stance phase (114) due to impaired sensorimotor integration caused by the vestibular-cerebellar lesion. Notably, DBN frequency and postural imbalance were significantly reduced during walking compared to standing (*p* < 0.001), and the frequency of nystagmus during walking was further modulated in a manner that depended on the specific phase of the gait cycle (*p* = 0.015) (115). The attenuation of DBN symptoms may result directly from the locomotor activity itself, reflecting selective central and/or peripheral suppression of sensory feedback during locomotion. Theoretical approaches (116, 117), clinical observations (118, 119), and experimental evidence (120, 121) suggest the presence of a predictive feedforward regulation of gaze and postural stability during locomotion. Thus, efference copies of the locomotor command could also counteract the destabilizing vestibulo-cerebellar drive that has been suggested to cause central oculomotor and postural impairments in DBN patients.

Diplopia, internuclear ophthalmoplegia (INO) and/or skew deviation (14, 122), when present, are due to concomitant involvement of the MLF (27, 55).

Interestingly, patients with DBS may have worse symptoms in the morning and instead improve during the rest of the day (99), may improve after resting in an upright position (123) and after being left in the dark (108).

## Associated oto-neurological signs

Due to the presence of spontaneous vertical nystagmus, smooth pursuit and optokinetic reflex in the vertical plane are altered, configuring a “false” (extrinsic) alteration of these reflexes. On the other hand, the alteration of smooth pursuit in the vertical plane is greater than would be expected from spontaneous nystagmus alone and from the deficit gain of smooth pursuit and the optokinetic reflex. Such a finding would seem to indicate a more widespread and “real” alteration in the central vestibular pathways.

In addition, due to these extensive changes, gaze-evoked and rebound nystagmus in the horizontal plane can often be observed. These findings are probably due to the close topographical and functional relationship between the cerebellar pathways for vertical movements and those for horizontal movements, especially those originating from the flocculus and vermis (108). In these cases, smooth pursuit may be apparently normal when the target is moving upward, the slow phase of nystagmus being in favor of smooth eye movement.

In some patients with DBS, unilateral or bilateral deficit vestibulopathy, especially at high frequencies, may be observed.

In addition to DBN, it is not uncommon to observe cross-coupled (perverted) nystagmus during caloric stimulation. The latter can be attributed to a disruption of the nodulus-uvula complex, the structure physiologically responsible for conjugating the plane of the reflex response with the plane in which the stimulus occurs (32).

Finally, the association of DBN with polyneuropathy and mono- or bilateral deficit vestibulopathy, especially at high frequencies, is not uncommon. The latter association suggests a common pathogenesis (1, 124, 125) probably involving calcium channel abnormalities, particularly in Purkinje cells.

## Etiology

Approximately 40% of DBS have an unknown cause and are considered idiopathic primary forms. In the remaining cases, secondary forms, a sporadic (i.e., vascular, malformative, degenerative, neoplastic, infectious, paraneoplastic, toxic, deficient, and iatrogenic) or genetic cause can be recognized. Various sites and structures may be involved in these secondary forms:

1. Cerebellum: flocculus/paraflocculus (FL/PFL), vermis, cerebellar hemispheres.
2. Brainstem: PMT neurons, pontine ventral tegmental tract (VTT).
3. Cervicomedullary junction (CMJ).
4. Foramen magnum region, and atlanto-occipital joint.

The most frequent causes of acquired forms of DBS are:

1. Vascular pathologies such as:
  - ischemic and hemorrhagic stroke (1, 88, 122, 126–129);
  - arterial malformations as: dolichoectasia of the vertebrobasilar circulation (130–132);
  - compression of the vertebral artery (133).
2. Inflammatory and autoimmune diseases such as:
  - optic neuropathy (134);
  - demyelinating pathologies (89, 122, 135–137);
  - autoimmune thyroiditis and Hashimoto’s encephalopathy (138);
  - celiac disease (139–142);
  - anti-GAD antibodies associated with DBN, idiopathic cerebellar ataxia, stiff-person syndrome, epilepsy and limbic encephalitis (143);
  - anti-NMDA receptor encephalitis (144).
3. Infectious diseases such as:
  - cephalic tetanus, which inhibits GABAergic Purkinje cells (145);
  - herpes encephalitis affecting brainstem or flocculus (146, 147);
  - West Nile virus encephalitis affecting the CMJ (148);
  - chronic human T-lymphotropic virus- 1 (HTLV-1) infection causing degeneration of cerebellar vermis (149).
4. Degenerative pathologies such as:
  - multiple system atrophy (38);
  - amyotrophic lateral sclerosis (150);
  - motor neuronopathy (151).
5. Malformative conditions such as:
  - Arnold Chiari disease (7, 152–155) (reported in 4–7% of patients with type I Arnold Chiari malformation);
  - syringobulbia (14, 122, 156, 157);
  - platybasia (8, 122, 158, 159);
  - “foramen magnum syndrome” (160).
6. Paraneoplastic syndromes include small cell lung cancer (with anti-CV2/CRMP5, anti-Hu, anti-Zic1/4 antibodies), ovarian and breast cancer (with anti-Yo antibodies), testicular neoplasms (with anti-Ma antibodies), thymoma (with anti-CV2/CRMP5 antibodies) and Hodgkin’s lymphoma (with anti-Tr antibodies) (161); anti-Yo and anti-Tr antibodies syndromes involving cerebellar degeneration (162–165), anti-Ma antibodies encephalitis (166, 167), anti-HU antibodies brainstem encephalitis (168).
7. Toxic conditions that may be attributed to toluene exposure and, above all, to ethanol chronic abuse (169, 170), the latter resulting in vitamin B1 deficiency and Wernicke’s encephalopathy (171, 172). Interestingly, also in non-chronic alcoholics forms, an excessive alcohol intake can cause reversible, short-lived DBN (173, 174).
8. Deficiency-type cerebellar disorders, which include vitamin B12 and Magnesium deficiency, a modulator of excitatory Ca2^++^ NMDA channels in cerebellar granule cells, which is also often associated with a lack in vit B1 (175–180).
9. Iatrogenic form due to:
  - antiepileptic drugs such as phenytoin (181), carbamazepine (even in therapeutic range) (182), lamotrigine (183), pregabalin (184). These drugs inhibit voltage-gated sodium channels Nav1.1 and 1.6, which are heavily expressed in Purkinje cells (185);
  - antiarrhythmic agents, such amiodarone (186);
  - intravenous opioids morphine, fentanyl, pethidine and piritramide (187–190), as well as intrathecal morphine (191). These drugs may generate a DBN by virtue of the agonism of m-opioid receptors in the cerebellum inhibiting the inhibitory cerebellar projections to the superior vestibular nucleus;
  - gastric acid suppressants such as ranitidine (192);
  - lithium toxicity is only rarely a cause of DBN, and no clear mechanism has been elucidated (193–197), such as quietiapine fumarate and estazolam (198).
10. Other possible causes as head trauma, intracranial hypertension, hydrocephalus (199); vestibular migraine (199–201) and tumors, also including metastasis (202–205).
11. Complex hereditary neurodegenerative disease such as:
  - Spinocerebellar ataxia type 6 (SCA6) (206–209);
  - Episodic ataxia type 2 (EA2) (210–213) and familial hemiplegic migraine type 1 (FHM1) (214–216), both caused by mutations in the CACNA1A gene, which encodes for voltage-gated calcium channel widely expressed in Purkinje and granule cells;
  - Idiopathic cerebellar degeneration such sporadic adult-onset ataxia and multisystem atrophy (1);
  - Familial episodic ataxia (213, 217, 218);
  - Ataxia- telangiectasia (219);
  - Mitochondrial Encephalopathy with Lactic Acidosis and Stroke-like episodes (MELAS) (220).

Tran et al. (221) have recently proposed the following useful etiological classification:

1. DBN because of structurally evident pathology;
2. DBN associated with specified non-structurally evident causes;
3. Idiopathic DBN, which includes patients with:
  - “pure” DBN, without other signs or symptoms except for the oculomotor disorders associated with floccular disorders (i.e., impaired smooth pursuit, optokinetic reflex and/or lack of visual suppression of the VOR);
  - “cerebellar” DBN (i.e., DBN associated with cerebellar ataxia or dysarthria);
  - “syndromic” form (i.e., DBN associated with floccular signs/symptoms, gait abnormalities, limb ataxia, dysarthria, dysmetria, dysdiadochokinesis, tremor, hypotonia, and so forth).

In the “syndromic” form, other authors also include at least two of the following: bilateral vestibulopathy, cerebellar signs and/or polyneuropathy, the latter reflecting multisystem neurodegeneration or channelopathy (1).

## Genetic aspects

To explain the genesis of DBN, some genetic aspects that can alter the functioning of cerebellar cells, and in particular Purkinje cells, have also been studied.

For example, adult murine CACNA1A mutants, in which P/Q channels present in cerebellar Purkinje cells and responsible for calcium currents are particularly reduced, show an upward displacement of the eye globes of the eye and a reduced high-frequency VOR gain. This finding supports that a channelopathy may be the underlying cause in some cases of idiopathic downbeat nystagmus. The efficacy of a potassium channel blocker, such as 4-aminopyridine, in reducing downbeat nystagmus and improving horizontal and vertical VOR gain would support this hypothesis. The appearance of the signs only in adult mice would also suggest that a genetic polymorphism in the CACNA1A gene predisposes to the DBS, which would occur because of age-related degenerative phenomena (222–224).

Other interesting information comes from chromosome 5q14.1, which contains the genes DHFR (essential for folate metabolism) and MSH3 (involved in cell cycle regulation, apoptosis, and genome stability). Disruption of these genes is responsible for cerebellar disorders (225–227).

Finally, it is important to remember the role of the fibroblast growth factor 14 (FGF14) gene, located on chromosome 13, and highly expressed in Purkinje cells, which represent the only output of the cerebellar cortex. FGF14 modulates the intrinsic excitability of cerebellar Purkinje neurons (228) positively regulates voltage-gated KCNQ2/3 potassium channels (Kv7.2/7.3), voltage-gated sodium channels (which have nine well-characterized isoforms: NaV1.1-NaV1.9) (229) and presynaptic Ca^2+^ voltage-gated channels. A distinctive feature of Purkinje cells is the robust expression of Nav1.6-encoded sodium channels, which underlie the “resurgent” sodium current that is critical for maintaining the characteristic high-frequency burst of these cells. More than 80% of Fgf14−/− Purkinje cells are quiescent and do not repeatedly activate in response to excitatory stimuli. Immunohistochemistry revealed reduced expression of Nav1.6 protein in Purkinje Fgf14−/− cells. These observations suggest that FGF14 is necessary for normal expression of Nav1.6 in Purkinje neurons and that its absence, by altering the expression of Nav1.6 channels, impairs spontaneous and tonic repetitive activation of Purkinje cells. The recent identification of an association between a variation in intron 1 of the fibroblast growth factor 14 (FGF14) gene and idiopathic DBN in a genome-wide association study (GWAS) (225) and of a dominantly inherited (GAA)_≥250_ repeat expansion in intron 1 of FGF14 as the cause of spinocerebellar ataxia 27B (SCA27B)/GAA-FGF14 ataxia (230, 231), a late-onset slowly progressive cerebellar syndrome that is frequently associated with DBN (230, 232) provided evidence that FGF14 (AAA) repeat expansions, including (GAA)_200–249_ and (GAA)_<200_, are a monogenic cause of idiopatic DBN syndrome (230, 232–235). The frequency of FGF14 (GAA)_≥250_ expansions in DBN syndromes is 56% (42/75) for DBN plus additional isolated cerebellar ocular motor signs, 72% (23/32) for DBN plus cerebellar ataxia, 30% (17/56) for DBN plus cerebellar ocular motor signs and/or ataxia and extracerebellar features, which included bilateral vestibulopathy (BVP) and/or polyneuropathy, and 0% (0/7) for pure DBN (235). The unexpected high frequency of FGF14 (GAA) ≥250 repeat expansions in patients with a predominantly sporadic non-ataxic DBN presentation may be due to hte fact that DBN is a milder phenotypic presentation of GAA FGF14 disease, in which overt cerebellar ataxia and other multisystemic involvement can be absent or, in some cases, develop later in the disease course. The identification of additional floccular/parafloccular cerebellar ocular motor signs in all (GAA)_≥250_-FGF14 patients with DBN, which may present in isolation early in the disease course, up to 8 years prior to the development of gait impairment, indicates that the basic dysfunction arises from this cerebellar region and that pure DBN is an uncommon manifestation in GAA-FGF14 disease. Finally, DBN plus additional isolated cerebellar ocular motor signs but without overt ataxia, despite long disease duration (up to 16 years), raises the possibility that GAA-FGF14 disease may remain limited to the cerebellar ocular motor system without broader cerebellar involvement in a subset of patients. For these reasons, genetic testing for FGF14 GAA repeat expansion should become part of the diagnostic work-up of patients with idiopathic DBN (235). Another interesting aspect, in these patients bilateral vestibulopathy (BVP) has a low frequency occurrence of (only 10% of cases), is a late feature in GAA-FGF14 disease compared to cerebellar ataxia, developing on average more than 10 years after disease onset, is significantly less frequent in (GAA)_≥250_-FGF14 compared to (GAA)_<200_-FGF14 DBN syndromes, and tends to remain relatively mild in GAA-FGF14-related DBN syndromes despite prolonged disease duration. Moreover, impairment of the visual fixation suppression of the VOR is most strongly associated with and predictive of (GAA)_≥250_-FGF14 DBN and (29) (GAA)_200–249_-FGF14 than (GAA)_<200_-FGF14 patients (235).

What has just been reported justifies the symptomatic benefit of 4-aminopyridine, a blocker of mainly Kv1 (A-type) potassium channels, which is the drug of choice for the treatment of downbeat nystagmus because it acts precisely by increasing the excitability of CPs43 and restoring the neuronal firing precision of Purkinje neurons. The efficacy of 4 aminopyridine is in fact demonstrated in a mouse model of ataxia that mimics SCA6 (57, 236), in episodic ataxia type 2 (EA2) and in GAA-FGF14 ataxia (SCA27B), which are also characterized by DBN (232). Particularly, response to 4-AP represents a strong predictor of (GAA)_≥250_-FGF14 and (GAA)_200–249_-FGF14 status in patients with idiopathic DBN (positive predictive value, 89%).

## Conclusion

An important finding most often indicative of central vestibular dysfunction is the DBN whose presence is often included in a currently well-characterized clinical syndrome.

The diagnostic significance of this finding has only recently been widely understood as research into its origin and pathophysiology has progressed. We now know in sufficient detail the neural structures responsible for the delicate control of eye movements in the vertical plane and can therefore focus our attention on these structures when confronted with a DBN.

Nowadays therefore, DBN is to be considered one of those highly localizing pathologic findings, and its recognition should first lead to detailed neuro-radiologic studies to identify the most common structural chronic or acute causes of this sign.

However, DBN can also hide subtle causes that can only be identified through specific hematologic and clinical studies, which must be known because, even in these special cases, a state of diagnostic and therapeutic urgency can be realized.

Sometimes, to correctly define a DBN and its syndrome, it is also necessary to think about performing specific genetic studies aimed at detecting diseases of a neuro-degenerative nature.

Thus, DBN and DBNS therapy today can in some cases be causal, i.e., aimed at eliminating the triggering cause (as can be the case especially with toxic, metabolic, deficiency, autoimmune forms), but when this is not possible, it can also be symptomatic, i.e., aimed at reducing the effects that the presence of this sign can cause.

The aim of our work was to provide a comprehensive and as complete as possible picture of DBN and to give a possible explanation of the different functional aspects observable in the presence of DBN. Therefore, this paper may be a useful tool for the specialist who finds himself dealing with these patients in any evaluation context, but also for the clinician who finds himself dealing with such a sign and pathology even in a non-specialist context, such as in a first aid environment.

## Author contributions

VM: Conceptualization, Supervision, Writing – original draft, Writing – review & editing. BG: Conceptualization, Supervision, Writing – original draft, Writing – review & editing. GV: Conceptualization, Supervision, Writing – original draft, Writing – review & editing. MF: Conceptualization, Supervision, Writing – original draft, Writing – review & editing. AF: Conceptualization, Supervision, Writing – original draft, Writing – review & editing. VP: Conceptualization, Supervision, Writing – original draft, Writing – review & editing.

## Supplementary material

SUPPLEMENTARY VIDEO S1 AS, a 36-year-old female, presented for observation complaining of mild gait ataxia and imbalance aggravated by exertion, brief episodes of vertigo, difficulty observing moving objects, and intention tremor for approximately 6 months. Rare episodes of diplopia had also been present for several years. Neuro-otologic evaluation revealed a spontaneous downbeat nystagmus of low amplitude and frequency, not inhibited by fixation, accentuated by eccentric lateral gaze but especially by the prone position. The latter finding was particularly suggestive. The subsequent neurological examination revealed hyperreflexia and extensor plantar responses. Molecular genetic testing performed approximately 2 months later revealed spinocerebellar ataxia type 6. The patient's mother is also affected by SCA6.

SUPPLEMENTARY VIDEO S2 AC, a 76-year-old female, presented for evaluation of positional vertigo, which had started about 15 days earlier and was associated with imbalance, but more importantly with uncontrollable vomiting. For these reasons, the patient had already undergone a cranial MRI, which was normal. At the time of the visit, the neuro-otological evaluation was negative, so she was advised to return in case of a new acute phase. Three days later, the patient returned for evaluation of very severe imbalance and tremor. Neuro-otologic evaluation revealed intense spontaneous downbeat nystagmus. A biochemical blood test revealed a decrease in serum magnesium (Mg) level (0.2 mg/dL; normal range 1.7–2.5), resulting in a deficiency-type cerebellar disorder. Mg supplementation (400 mg/day, orally) was initiated, with rapid improvement of the imbalance and tremor. Fifteen days later, the patient was asymptomatic, and the blood magnesium level was 2.05.
